# Selections

**Published:** 1816-09

**Authors:** 


### PART III.

### SELECTIONS.

—— Factorum est copia nobis.

### *Case of Poisoning by oil of Vitriol (sulphuric acid) successfully treated.**

"In the seventh volume of Hufeland's Journal, is recorded the case of a country-man, aged 20, who took by mistake, instead of a stomachic medicine, sixty drops of oil of vitriol, and notwithstanding judicious treatment, died on the seventh day."

"I therefore, hasten to lay before the medical public, the history of a similar case of poisoning, which appears to me, on several accounts, worthy of communication. These are the delicate and irritable state of the patient; the quantity of the poison swallowed; the successful issue of the case; and lastly, the hope that it may operate as a warning against future mistakes of this nature."

"A woman, aged 38, had been subject, from the month of May, to great debility of the nervous system, in consequence of which she was so agitated by any disagreeable incident as to suffer slight convulsions. On the morning of the 9th of July, she was alone in her chamber, and felt an uncommon weakness of the whole body; whereupon she thought to reach some stomachic drops from a cupboard; but, unluckily, took hold of a glass containing oil of vitriol for a chemical match bottle; and half filling a large meat spoon with the liquid, swallowed it. Scarcely had she discovered the dreadful mistake, when she seized another glass in which was decoction of Peruvian bark, and took a spoonful of it. The daughter, who was in the next room, and observed the distressing accident, immediately ran for help. Previously to my arrival, which soon took place, milk and water had been administered, and violent vomiting was the consequence."

"Words are wanting to describe the dreadful situation in which I found the patient. There were indescribable anxiety; an intolerable sense of burning in the whole cavity of the mouth and pharynx; unquenchable thirst; violent efforts to vomit; convulsions. The patient indeed had lost all consciousness; and, whenever somewhat revived, expressed only a wish that death would terminate her sufferings.

* *Geschichte einer glucklich geheilten vergiftung durch vitriolol. Von H. H. Dr. Memminger. Journal der pratischen Heilkunde. Von Hufeland, &c. November,* 1815.

"I immediately directed cold applications to the breast and throat, sinapisms, injections of milk. I gave internally mucilage of gum arabic, syrup of diacodium, oil of sweet almonds, and a decoction of bark, valerian, and mallow; for common drink, almond milk, and barley-water. In the afternoon, my patient had so far recovered, that I could inspect the cavity of the mouth. The tongue was thickly covered with an ash grey slime; the lower lip excoriated; the uvula, tonsils, and velum palati, every where highly inflamed, but not corroded by the acid. The greatest pain experienced in deglutition, was in the neighbourhood of the thyroid gland. Here the patient thought she felt a knot; and here also were the most excruciating pains whenever medicine or other fluid was passing into the stomach: indeed the greatest efforts were required to force them down, and they were frequently rejected.

"On the second day, a separation took place from the throat, of some black shining substances, which, viewed under a microscope, had the appearance of burnt flesh. Third day. An immense quantity of phlegm was yet thrown up: it incommoded the patient very much, and so alarmed her, that she dare not sleep from the dread of suffocation. The former remedies were continued. Fourth day. The tongue was cleaner; the thirst and sense of burning diminished; appetite returning; but the deglutition of both solids and fluids troublesome. Fifth day. The menses should have appeared, but did not; and, at this period, all the complaints had been, for a long time, invariably aggravated. Deglutition was more difficult; the pulse fuller; thirst and heat increased; and the inflammation of the velum palati again considerable. Not daring, for several reasons, to employ venæsection, I now directed leeches to be applied to the throat; and they were productive of speedy relief. But menstruation did not take place. Sixth day. The patient had some sleep during the night, and found herself more comfortable; and particularly relieved by a sweat which had broken out. In order to cleanse the throat, I directed a mixture of honey of roses, syrup of mulberries, and essence of myrrh; but she could not bear its employment, as it was productive of too much irritation and pain. I was, therefore, compelled to have recourse again to the mucilaginosa. Seventh day. Great as the hope of recovery had been, it was, this day, much diminished. The pulse was uncommonly small and rapid; the patient dejected, and again so despairing of recovery, that she gave directions about her funeral. Bitter infusion prescribed. Eighth day. So much recovered that she expressed a wish to take food. Ninth day. The last night was the best she had hitherto passed. The bitter infusion was continued, and volatile liniment rubbed in upon the throat. Tenth day. The menses reappeared; and, from this time, restoration proceeded so rapidly, that I think it needless to detail any further particulars. At the present day, (August 30th.) she is so perfectly recovered, as to leave no room for doubt or apprehension as to the event."

"I may yet, in conclusion, remark, that this incident, however unfortunate in itself, has been productive, in other respects, of happy consequences : since the patient, from that time, has experienced no return of her nervous fits : and thus is here verified the proposition : *nil nocet, quod non prodesse possit idem.*"

### *Case of Worms contained in the Urinary Passages, and discharged alive from the Urethra.**

"M. de Monciny, aged 50, of a mild and sprightly disposition, middle stature, constitution neither robust nor delicate, of a mixed temperament, but more sanguine than bilious, and engaged in a military life, was under the necessity of repairing to Middleburgh, in the island of Walcheren, as hospital paymaster. He remained eighteen months in that unwholesome climate. A few observations on the topography of this country may not be irrelavent. The city of Middleburgh is built upon a soil flat, marshy, and undrained, in the vicinity of the sea, and exposed to the influence of the tides. The only water drunk is rain water, collected in cisterns from the house roofs. If rain were frequent, this water, often renewed, might not be unwholesome ; but, on the contrary, it remains stagnant in the cisterns, and grows foul and pernicious in dry weather ; which must greatly augment the number of diseases of any army, stationed there. Every one knows that the mortality which the English experienced in the island, obliged them to evacuate it. The French also have sustained heavy losses while they occupied it; and especially during the prevalence of certain southerly winds.

"From the corruption of the water just mentioned, insects are engendered, and, among others, small and almost invisible worms, of which the inhabitants take little notice; especially the more opulent, who obviate their noxious effects by drinking good wine, tea, beer, spirituous liquors, and smoking tobacco.

"M. de Monciny, a sober man, little inured to the habits of the country, frequently drank the water as it was; yet sometimes he mixed it with wine, of which, however, the high price made him sparing.

"During his residence in this island, he suffered four febrile attacks. The first was of the continued type, and lasted almost three weeks;† the second, quotidian, but ten days ; the third anomalous ; and the fourth a regular tertian, seized him on the road, in his departure from the country.

---

* *Observations sur des vers contenus dans les voies urinaires, et rendus vivans par l'uretre. Par M. Duchateau, Docteur en Medecine. Bulletin de la Societé Medicale d'Emulation. No. III. Mars,* 1816.

† We have ventured to put *weeks*. The word in the original is *septenaries,* seven *years,* evidently a misprint. Edit.

"All these attacks were attended with violent pain in the loins, right kidney, and urethra, and in the corresponding iliac region. Considerable *hæmaturia* appeared at the same time. The blood, almost always voided with the urine, was bright red as in active hæmorrhage. The patient felt pain when certain foreign bodies passed through the neck of the bladder; he *believed* them to be coagula of blood; but never had the curiosity to examine.

"Last November, my patient was recalled by government to take a situation in the South of France. This summons was, on all accounts, most welcome. During the first day of his journey, the coach fatigued him greatly. On that evening, he was seized a fresh with the pain in the right kidney and course of the ureter, extending to the bladder. He was compelled to quit the vehicle, and go to an inn. Here he experienced a shivering fit, of two hours, with tremor, followed by a feverish paroxysm, which lasted eight hours. At its close, blood was again voided with his urine. On the morrow, being the day of remission, he found himself better and proceeded to Paris. He was seized, on his arrival, with a second fever fit; of which he had felt the prelude two hours previously, He had the same pains as during the first paroxysm; but voided no blood.

"On the evening of the 4th of December, 1812, I found M. de Monciny very feverish. In my examination, I discovered tension about the liver, with turgescence and pain, as well as in the right lumbar region. Descending along the anterior margin of the ileum, and more deeply in the iliac fossa of that side, the pain extended to the neck of the bladder. Little urine was voided; and that produced a sensation of heat as it traversed the urethra. I requested that what was made during the night, might be kept till morning. It was scanty, in consequence of the sweats which terminated the paroxysm; high-coloured, and had deposited a reddish sediment, partly mucous, partly lateritious.

"On the morning of the 5th, my patient was tolerably well, not very feeble; pulse calm and regular; skin moist; tongue sooty. He felt disgust for food: his stools were scanty.

"I took care to profit of the day of remission to apply eight leeches to the anus, and to place the patient, during twenty-five minutes, on warm water: I directed a glyster previously to this application, diluent drinks, and fomentations in the course of the pain, notwithstanding that it was less severe than during the paroxysm.

"6th. Morning. My patient was much relieved: the heat and tension of the affected parts being diminished; but, as he had felt slight spasms in all the limbs during the night, I prescribed a sedative julep.

"7th. The third paroxysm came on, this morning, at the usual hour. The shivering had been less long and violent; the heat less intense; the sweating moderate; the urine more copious and light-coloured; but the fur of the tongue and disgust for food were aggravated. Attending to this indication, I prescribed a vomit which produced an evacuation of brown-yellow bile, a little greenish bile, and three stools of a diluted brown matter. The same regimen was continued. The pains were now become trifling; the urine more copious and saffron-coloured, always depositing some flakes with a little sediment of an indeterminate reddish colour: it was almost destitute of smell.

"8th. Morning. My patient had passed a comfortable night, and hoped to miss the evening paroxysm; but in this he was deceived: it returned two hours earlier. The shivering was of shorter duration, and the hot fit terminated only at three in the morning.

"9th. I prescribed tamarind-water to open the bowels, as the tongue, though not dry, was always furred: the urine unchanged. Broth was, several times, taken during the day and night.

"10th. Same as yesterday: tamarind water repeatedly taken."

"11th. The paroxysm of yesterday came on two hours earlier than its predecessor; but was more short and weak. Four stools were procured by a laxative.

"12th. Going on well. More nourishment. I felt disposed to terminate the fever by the exhibition of cinchona.

"13th. My project was frustrated. The paroxysm had been as strong as ever; the shivering violent; the pains of the kidney and urinary passages more severe. I learned that the patient had voided a chamber-pot-full of blood and coagula, which had caused great pain in passing the urethra. To my regret, the vessel had been emptied. My patient was more feeble, but easier. The paroxysm was now on the decline. The tongue and skin were dry; the countenance somewhat dejected. I repeated my former examination, and found only the remains of excitement, and a little tension in the lumbar and abdominal regions of the affected side. This recurrence of hæmaturia almost at the decline of the malady alarmed me. I began to suspect the existence of calculus; and that the hæmorrhage had been determined by a fragment of it. I was about to propose a consultation when the patient called for his pot. After making water on his knees, he claimed my attention to a coagulum of blood, which had just passed with great pain. About half a pint of urine or blood had been discharged. Some bright-red blood was on the margin of the vessel. The fluid was turbid: after standing some minutes, I decanted it carefully into another pot. Something at the bottom attracted my notice. Examining more closely, I distinguished a *living worm.* Placed upon a plate with a little cold water, it moved about. Ignorant then that any such phænomenon had ever been observed, I could scarcely persuade myself that this worm had issued from the urethra.

"The worm was of a red brown colour; about four inches long, large as a *lumbricus;* about a line in diameter from one of the extremities to its middle: the other was thread-like, flat and pointed at the extremity. The large end presented a head flattened below, like that of the leech, and suckers smeared with blood. This head terminated in a kind of trunk or antenna; having in the middle of the body an appendix or species of vermicular cord. By means of a microscope, I distinguished several rings in the thickest part of the body.

"Diluents were again prescribed for my patient, and a julep to soothe irritation. When convinced that he had voided this worm, he told me that, if this were the case, he had voided many others both at Middleburgh and Paris.

"14th. I found my patient more calm. All the symptoms were much mitigated since morning. He had again, during the night, discharged a potful of urine mixed with blood : it was less red from having been five or six hours in the pot. On pouring it off, I found at the bottom of the vessel, a second live worm, like the first, and besides, a very small one, of the thickness of a thread, and an inch in length. It was very lively, and when seen in a microscope, no doubt could be entertained of its existence or structure, like that of the two larger.*

"M. de Monciny, from this time, had a rapid and almost uninterrupted recovery. He twice or thrice experienced a return of the febrile paroxysm and nephritic pain : and, on the twenty-first, voided a potful of bloody and offensive urine, without worms, but containing, at the bottom, a membranous and spongy substance, thickish, brown in colour, and of a fœtid smell. By the administration of cinchona, combined with the mineral water of Bareges, M. de M. was soon perfectly restored, and able to repair to his destination in the South of France."

The "Reflexions" of M. Duchateau, our limits will not, at present, allow us to transcribe ; but this is not of much consequence ; as we intend, ere long, to present a sketch of the history of urinary worms, in which every thing, worthy of observation, afforded by the paper of the French physician, will be duly noticed.

---

### *Case of Polydipsia, by Mr. Ware, of Cambridge, North America.*

The subject of the following account, is a young man, named James Webb, now living in the town of Hingham, in this state. Having been informed of his singular case, I went in company with Mr. Norton, librarian of Harvard University, to ascertain its nature, and the truth of the circumstances related concerning him. During our visit, the following facts were collected from his own account of himself, which were confirmed by the testimony of the persons with whom he now lives, and as will be presently stated more particularly, by that of others with whom he has formerly lived.

He was twenty years of age in the month of Oct. 1814. His

---

* Two of these worms were preserved by M. Duchateau, and presented for inspection, to the members of *La Société Medico-Practique*. Editors.

appearance is that of firm health ; his complexion dark and ruddy ; he is short, thick, rather sturdy, and, except his preternatural thirst, has never been afflicted with disease. At present, the quantity which he habitually drinks in twenty-four hours, amounts to three pailsful, or six gallons. This is necessary, not only to prevent the sensation of a tormenting thirst, but to preserve him in his ordinary state of health ; for when he abstains from his usual allowance, his head is affected, and he becomes dizzy, weak, and sick ; or, as he himself expressed it, " When I don't drink, it gets into my head." He finds himself obliged to drink at intervals of about an hour and a half or two hours, one or two quarts at a time. At night he places a pailful of water at his bed-side, the whole of which he requires before morning, waking whenever it becomes necessary. He has sometimes taken to the amount of a gallon at once, without experiencing any bad effects. He drank two quarts in our presence, having taken one but fifteen minutes before. He swallows with great eagerness and the appearance of satisfaction, drinking a quart in the time that a common person would a quarter of that quantity. He uses water directly from the well, even in the depth of winter, and avoids mixing any thing with it, especially any kind of spirituous liquors, which he dislikes. Its coldness causes no inconvenience, except occasionally a slight chill. He has no recollection of the time when this habit commenced, but has been told by his parents that it was in infancy, and soon after birth. The quantity which he now drinks, does not, he thinks, differ materially from that which he drank at nine or ten years old. He has several times endeavoured to break off the habit, but has always suffered from the attempt in the manner above-mentioned. His appetite for food is not remarkable ; the persons with whom he lives merely observing, that he was a hearty eater. His meat and drink at meals, are like those of the persons with whom he lives. His pulse during our visit, was full, strong, and remarkably infrequent, not exceeding, at any time, 56 pulsations in a minute, and being sometimes so few as 45. It varied as follows :

Some time after drinking, 56. Fifteen minutes after drinking, and just before drinking again, 50. Immediately after drinking two quarts, 45.

The temperature of the atmosphere has no influence on his thirst, since he requires the same quantity of water in the warmest, as in the coldest weather. He had an uncle who was formerly affected in the same way, although not to an equal degree. He served in the army during the revolutionary war, and was said to have died in consequence of being in a situation where water was not to be obtained.

As would be supposed, these extraordinary quantities of fluid are wholly carried off by the kidnies, and affect the secretion of no other part. His urine, he thinks, equal in quantity to the whole of the water which he drinks. The qualities of the secreted fluid, I had no opportunity of examining. He perspires very little, and his fæces are of the usual healthy consistence.

This account is confirmed by Messrs. Wilder and Hersey, two persons with whom he formerly resided. With Mr. Wilder, he lived some time at about the age of nine or ten years, and his relation agrees with that of Webb in every particular, especially as to the quantity of water drunk during twenty four hours.

With Mr. Hersey, Webb lived from fourteen to eighteen years of age. He thinks that the quantity increased while he was with him, and feels confident, that during the latter part of the time he drank as much as four pailsful, or eight gallons daily, and has known him to drink a gallon at a time. He never knew him suffer from want of water but once, when away from home where none was to be obtained; he looked pale and said he could not live ten minutes longer, but was relieved by drinking. He used to shudder for ten or fifteen minutes after drinking, so that his teeth would chatter. His health and appetite were good. He used no spirit. Mr. Hersey says, that he has understood from the person with whom he lives now, that the quantity has diminished since Webb has been with him. *New England Journal of Medicine and Surgery*, 1815.

